# Automated PDF highlighting to support faster curation of literature for Parkinson’s and Alzheimer’s disease

**DOI:** 10.1093/database/bax027

**Published:** 2017-03-27

**Authors:** Honghan Wu, Anika Oellrich, Christine Girges, Bernard de Bono, Tim J.P. Hubbard, Richard J.B. Dobson

**Affiliations:** 1 Department of Biostatistics and Health Informatics, King's College London, De Crespigny Park, Denmark Hill London SE5 8AF, UK; 2 School of Computer and Software, Nanjing University of Information Science and Technology, 219 Ningliu Road, Nanjing, China, 210044; 3 Farr Institute of Health Informatics Research, UCL Institute of Health Informatics, University College London, London Gower Street, WC1E 6BT, UK; 4 Department of Medical and Molecular Genetics, King's College London, Guys Hospital, Great Maze Pond, London SE1 9RT, UK

## Abstract

Neurodegenerative disorders such as Parkinson’s and Alzheimer’s disease are devastating and costly illnesses, a source of major global burden. In order to provide successful interventions for patients and reduce costs, both causes and pathological processes need to be understood. The ApiNATOMY project aims to contribute to our understanding of neurodegenerative disorders by manually curating and abstracting data from the vast body of literature amassed on these illnesses. As curation is labour-intensive, we aimed to speed up the process by automatically highlighting those parts of the PDF document of primary importance to the curator. Using techniques similar to those of summarisation, we developed an algorithm that relies on linguistic, semantic and spatial features. Employing this algorithm on a test set manually corrected for tool imprecision, we achieved a macro F_1_-measure of 0.51, which is an increase of 132% compared to the best bag-of-words baseline model. A user based evaluation was also conducted to assess the usefulness of the methodology on 40 unseen publications, which reveals that in 85% of cases all highlighted sentences are relevant to the curation task and in about 65% of the cases, the highlights are sufficient to support the knowledge curation task without needing to consult the full text. In conclusion, we believe that these are promising results for a step in automating the recognition of curation-relevant sentences. Refining our approach to pre-digest papers will lead to faster processing and cost reduction in the curation process.

**Database URL:** https://github.com/KHP-Informatics/NapEasy

## Introduction

Parkinson’s and Alzheimer’s disease are the two most commonly occurring neurodegenerative disorders (1). According to the Alzheimer’s Association Report (2), 1 million new cases of Alzheimer’s per year are expected to be diagnosed by 2050. Similarly, numbers of Parkinson’s disease sufferers are expected to rise to between 8.7 and 9.3 million by 2030 (3). With the number of people being affected by both diseases constantly rising, interventions are needed . In order to develop these interventions, a common understanding of the underlying causes is needed as well as knowledge about the pathology of these diseases over time.

In current medical practice and clinical research, patients are often assessed using neuropsychometric tests such as the Movement Disorder Society sponsored revision of the Unified Parkinson’s Disease Rating Scale (MDS-UPDRS) (4) or the Montreal Cognitive Assessment (MoCA) (5). However, it is difficult to ascertain the affected brain regions from these scales without expensive medical procedures. Importantly, there are numerous empirical reports and case studies which correlate affected brain structures with performances on such neuropsychometric tests. These could be leveraged to further our understanding of the relationship between tests and brain anatomy (6–9) and with that, identify potential causes and track disease progression.

The need to improve our understanding regarding the relationship between brain structure and function in the context of neurodegeneration was one of the key drivers for the ApiNATOMY project (http://apinatomy.org/home) (10). Within this project, several lines of information were gathered manually through the curation of case studies, experimental research papers and reviews that report predominantly on Parkinson’s and Alzheimer’s disease (e.g. 11, 12). The types of data extracted from these papers included: (i) the aim or goal of the study; (ii) patient samples; (iii) what type of neuroimaging method was used in the study to examine the brain; (iv) which neuropsychometric tests or experimental tasks were implicated in order to confirm the presence (or absence) of neurological impairments and (v) significant results which correlated neuroanatomy with behavioural, cognitive or motor deficits. Such information was then abstracted to enrich ApiNATOMY’s knowledge base. Before the information could be abstracted the curator marked up the PDF file of a paper, highlighting those passages that were relevant for making the connection between test and neuroanatomy. This page (https://github.com/KHP-Informatics/NapEasy/wiki/Curation-Process) (Supplementary Document 1) presents a more detailed description of the sought-after information in the curation process.

In this study, we particularly focused on the first step of highlighting key sentences containing information of multifaceted importance as outlined in the previous paragraph, to ease the workload on the human curator. While this aim can be compared to deriving a summary of a paper, it goes beyond general automated text summarisation (13) in that the sentences used for our aim may not necessarily retain the most important sentences of the original document. However, methods to build so-called extractive summaries (selecting sentences that best represent the content of a text) (14) could potentially guide the automated highlighting process.

In particular, one important difference compared to automated text summarisation is that the number of highlighted sentences in our curation task varies across publications and could potentially be small or large. In our development data set, there are many cases where a large number of sentences were highlighted by the curator, e.g. the maximum number of highlighted sentences is 30 and there are >15 highlighted sentences in a quarter of them despite the varying length of the papers. In such cases, knowing the types of automatically highlighted sentences (e.g. it is a *finding* or *method* sentence) can effectively guide the human curator to look up information at the right places, e.g. the conclusions of a study are likely to be found in *‘finding’* sentences, while the experiment parameters and settings instead are expected to reside in *‘method’* sentences. Being able to immediately identify the relevant sentences, the curators can use their time more wisely. For example, one can check *‘goal’* sentences to assess the relevance of a paper before conducting any actual curation work. Similarly, checking ‘*finding*’ sentences helps a quick judgement on whether the conclusions reported in the study are worth further curation efforts.

Here, we implemented a method that used sentence-level linguistic features and spatial information across the entire publication to (a) automatically highlight sentences that matched the abovementioned five criteria and (b) further assign highlighted sentences with one or more types of *goal*, *method* and *finding*. We applied the methodology on two different data sets: one used for the development of the model and a second, to test generalisability. Using these data sets, we achieved a macro-averaged F_1_-measure of 0.53 on the development data set and of 0.32 on the test data respectively. As the evaluation relied on the automated recognition of manual curator highlights and could potentially impact on the performance of the algorithm, we manually corrected a small subset of the test data set. Using the corrected test data set for evaluation, we achieved a macro-averaged F_1_-measure of 0.51, which we believe to be a promising first step in the direction of automated curator support. A user based evaluation showed that the automatically generated highlights could already significantly facilitate the knowledge curation task in most cases. With these promising results, we believe that once improvements are made as outlined, the proposed approach can significantly speed up the curation process and consequently lower costs in projects such as ApiNATOMY.

### Related work

While not identical, the task of identifying important sentences for automated PDF highlighting can borrow ideas from automated paper summarisation. Existing summarisation techniques are either of extractive or abstractive (14), with the former assigning scores to individual sentences and based on these scores deciding whether the sentence should be included into the summary of the paper. While methods generating extractive summaries are relevant to the task at hand, our use case is more complex in that the sentences needing to be highlighted are not necessarily those that would best represent the content of the paper and appear in the summary. As pointed out in the introduction, the five-faceted criteria for sentence selection in our use case scenario is that it needs to be relevant for a curator to be able to judge on patient samples, neuroimaging methods and neuropsychometric tests, and correlations between neuroanatomy and behavioural, cognitive or motor deficits.

Due to the relatedness to extractive summaries, we briefly mention some of the existing approaches here. For example, Gupta et al. published an algorithm aiming to summarise papers by identifying lexically strongly connected sentences, using features such as term frequency, word co-occurrence, location and cue words (15). The authors report that there is only a deviation in performance of 8–12% between summaries generated through their algorithm and human curation. In Fattah and Ren (16) report about training different kinds of models (regression, neural networks, etc.) on features like positive/negative keywords, sentence centrality and similarity to title, relative sentence location and length. Using these features, the best performance with a precision of 61.78% on 100 English articles is achieved using a Gaussian Model and defining a requirement of 30% of text being represented in the summary. As a last example, Contractor et al. use argumentative zones (as defined by Teufel and Moens (17)), for the creation of summaries (18). Argumentative zones (e.g. Aim, Background, or Contrast) group sentences according to their rhetorical status with respect to the discourse of the paper (17). The authors report an improvement of 7% in F_1_-measure performance for full document summarisation using automatically identified argumentative zones (19), alongside other features such as verbs and sentence location.

All the approaches provided here for creating extractive summaries share the incorporation of lexical as well as location features, which we adopt in our approach. However, we note here that for our task it is not possible to pre-determine the number of sentences that need to be highlighted in the paper. This can be observed as the large variation of sentences highlighted in our development data set [see online materials (https://github.com/KHP-Informatics/NapEasy/blob/master/results/sentences_highlights.png (red line in the figure shows an arbitrary cut-off of 5% of sentences)]. The reason is that, in our scenario, whether a sentence should be highlighted is determined by whether it falls into (at least) one of the five types of information listed in the introduction section. The number of sentences that fit this criteria depends on the type of the publication (e.g. review papers tend to have more findings than research papers) and also the study/studies described there (e.g. some studies may use more tests than others).

Another line of relevant work includes approaches for classifying sentences in biomedical publications into Introduction, Methods, Results, and Discussion (IMRAD). The first automated sentence classification approach was reported by McKnight and Srinivasan (20), which was followed by a series of work (21–24). Although there have been some efforts to classify sentences in full-text biomedical articles (24), most of research efforts (20, 21, 22, 23) have focused on classifying sentences in the abstract of a publication. The commonly used features in these studies were bag of words (n-gram), linguistic features of verbs and sentence location. In this work our sentence classification was mainly derived from subject–predicate patterns. The performance of previous studies varied significantly. For example, in the studies above, F-scores ranged from 52 to 92%, which implies that the task challenge depends on the chosen corpus and specific task definitions. While sentence classification was one of the functional requirements of our task, our main goal in this work was to accurately learn a curator's highlighting behaviour. Consequently, the methodology proposed and evaluated in this paper was targeted to achieve a good automated highlighting model. This being said, our hypothesis was that a good sentence classification would improve the efficiency of curation tasks. Although our classification targets are not inline with IMRAD categories (see Supplementary Documents 2 and 3), whether IMRAD classification methods can be utilised to improve the curation task remains as an open question, which is out of the scope of this paper but certainly a good research question worth exploring in the future work.

More generally, natural language processing (NLP) and text mining technologies have been widely used to support curation tasks in both biomedical and clinical domains. For example, Moen *et al.* (25) conducted a study of automated text summarisation on clinical notes, which showed the high correlation between manual and automated evaluations, and consequently concluded that the less labour-intensive automated evaluations could be used as a proxy for human evaluations when developing summarisation methods. Karamani *et al.* (26) employed the Named Entity Recognition (NER) and Anaphora Resolution modules to help curation tasks, which showed 58% improved navigational efficiency and 74% better navigational utility. Similarly, Alex *et al*. (27) observed a maximum reduction of 1/3 in curation time (apart from the fact that more records could be curated) in NLP assisted curations. It is also worth mentioning community events like BioCuration conferences (http://biocuration.org/community/conferences/international-biocuration-meetings/) and BioCreative events (http://www.biocreative.org/events/), which have significantly helped advance technologies and synergise researches in this area. However, despite exciting and encouraging progresses, we could not find off-the-shelf tools/techniques that could be applied directly in our scenario. Apart from the specificity of our tasks, one reason might also be the scale of the challenge in turning successful techniques/methods into real-world applications as pointed out by (28, 29).

## Methods

**Figure 1.**
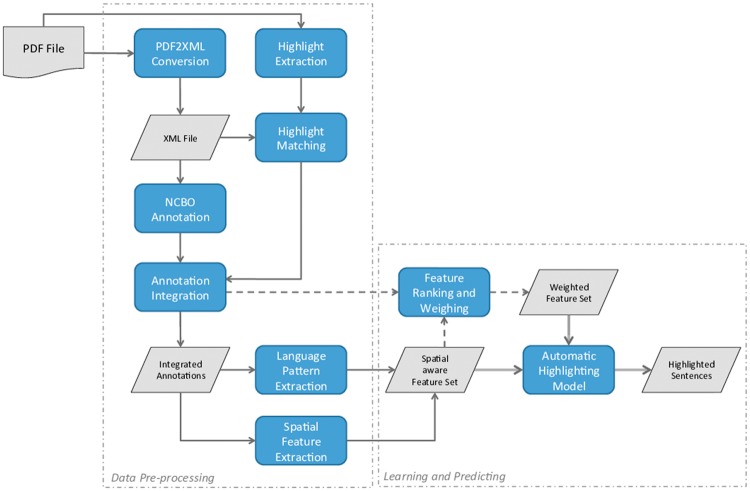
Illustration of the individual steps of the developed pipeline.

The work presented in this study aimed at generating automated PDF highlights that could be used for a curator to quickly assess patient samples, neuroimaging techniques, psychometric tests and potential correlations between neuroanatomy and behavioural, cognitive or motor deficits. We applied linguistic and semantic features as well as spatial properties to determine whether a sentence should be highlighted or not. Using PDF files as input data, we have developed a pipeline that includes several steps for data processing and sentence highlighting. The overall workflow of this pipeline is illustrated in Figure 1 and its individual steps are further explained in the following subsections.

### Input data

In this study, we investigated 275 full text papers that were manually curated and highlighted by a senior curator (author CG) for knowledge curation the ApiNATOMY project. From these 275 papers, only 241 papers could be converted into Extensible Markup Language (XML) files using Partridge (https://papro.org.uk/author/admin/) (30). The resulting collection of 241 papers was divided further into two sets: the *development* data set for developing our prediction algorithm (183 papers; 35k sentences, out of which 2213 were highlighted; 86 different journals) and the *test* data set for testing the suggested methodology (58 papers; 11.4k sentences, out of which 834 were highlighted; 33 different journals).

The PDF files were also processed with the Poppler Qt4 Python library (https://people.freedesktop.org/∼aacid/docs/qt4/ and https://pypi.python.org/pypi/python-poppler-qt4/) to extract the highlights that had been manually assigned by the senior curator to the PDF files. The extracted highlights were then matched to the sentences in the XML files using string matching. All the processing of the files was conducted using Python scripts that are provided for reference in the following online repository: https://github.com/KHP-Informatics/NapEasy.

### Linguistic and semantic features used for highlighting

In order to automatically highlight sentences for further curation, we used three different types of linguistic and semantic features: (i) cardinal numbers preceding a noun, (ii) named entities and (iii) subject-predicate patterns. Cardinal numbers were extracted by applying part-of-speech (POS) tagging (https://www.ling.upenn.edu/courses/Fall_2003/ling001/penn_treebank_pos.html) (31) as implemented in the Stanford parser (32). We considered everything that was labelled with the POS tag CD to represent a cardinal number further specifying a noun. For example, in the sentence ‘We further investigated 10 elderly patients’, 10 would be extracted as a cardinal number.

To allow for a broad recognition of named entities that are relevant to neuropsychometric tests and brain anatomy, we utilised two different named entity recognition systems: the National Center for Biomedical Ontology (NCBO) annotator (http://bioportal.bioontology.org/annotator) (33) and the named entity model implemented in the Natural Language Toolkit (NLTK) Python package (http://www.nltk.org/). The reason for incorporating two systems is that while the NCBO annotator covers over 300 ontologies provided through BioPortal (34), these ontologies do not cover all of the concepts needed for this specific domain. In particular, concepts for neuropsychometric tests and detailed brain anatomy as required by our use case scenario, cannot reliably be identified using the NCBO annotator.

The final group of linguistic features was again extracted using the grammatical structure of the sentences and the POS output of the Stanford parser applied to the sentences that have been manually highlighted by the curator. For the generalisation of this feature, we only considered sentences from papers in the development data set. Starting from the root of the parse tree, the first noun phrase or personal pronoun (indicated by labels NN or PRP) are extracted as the subject, while all the verbs in the following verb phrase (indicated by label VB) are extracted as the predicate of a sentence. Both are recorded for further, manual assessment. We note here that in some cases, sentences contain multiple subjects and/or predicates, in which case all subjects and predicates are extracted on a sentence-level.

**Figure 2.**
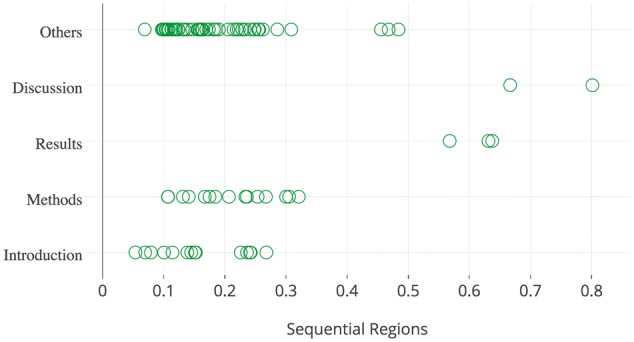
The spatial distribution of goal sentences extracted from the papers in the development data set.

When properly weighed (see Section 2.4) according to the type of the subject-predicate pair in relation to the structure of the paper, the extracted subject-predicate pairs were expected to be an effective predictor in determining which sentences should be highlighted. For example, aims (or goals) of the study are mostly expressed in the beginning of the paper, while method descriptions only follow in later stages (see Figure 2). For this purpose, all subject-predicate pairs from the development data were manually assigned (author AO) to one or more of the following three categories: (i) goal, (ii) finding and (iii) method. We note here that the judgment on the pairs was done without context, i.e. only subject and predicate were provided but no further contents of the sentences containing the subject and the predicate. As a consequence, the category assignment was conducted in the most inclusive way. This means that if a subject-predicate pair was expected to be used in complex sentence structures spanning more than one category, all covered categories were assigned. For example the subject-predicate pair ‘we used’ could be expected in a sentence such as ‘To study X, we used Y’. In this example, the first part of the sentence expresses an aim while the second describes the method to achieve this aim, thus causing ‘we used’ to be assigned to the goal and method category.

Subject–predicate pairs from the test set were not assessed manually to ensure an entirely independent evaluation set. However, experiments and observations revealed that subject–predicate pairs were very specific to the selection of papers and there was little overlap between the development set and test set. This means that the subject-predicate pairs need to be matched to those manually curated from the development data. Using an approach solely relying on exact matching would potentially mean missing many semantically identical patterns, which might impact the algorithm’s performance. To overcome this limitation, a similarity based matching strategy was implemented in our tool by making use of synonyms as defined by WordNet (35) for subjects and predicates. Algorithm  1 illustrates how WordNet was used to match a given subject–predicate pair to one of manually curated patterns.

### Spatial features incorporated for highlighting

One of the key factors in presenting scientific research in a paper is the layout. From an authoring perspective, there are often conventions or journal submission guidelines that guide the content arrangement. For example, the introduction section usually includes information on the background of the study, general discussions of the current state-of-the-art and often a brief summary of findings of the study. These layout arrangements are domain and language independent features that could complement, possibly even verify, the predictions of highlights based solely on language features, thus potentially correcting errors originating from the NLP. From a reading perspective, the spatial allocation of sentences takes effect through the reading habits of a human reader, e.g. the first appearance of similar sentences might be more likely to catch a sequential reader’s attention. Such a phenomenon might constitute an important factor in the decision of whether a sentence needs to be highlighted or not.

**Algorithm 1**. matching a given subject-predicate pair (sub, pred) to one of the manually curated subject-predicate patterns by making use of semantics represented in WordNet

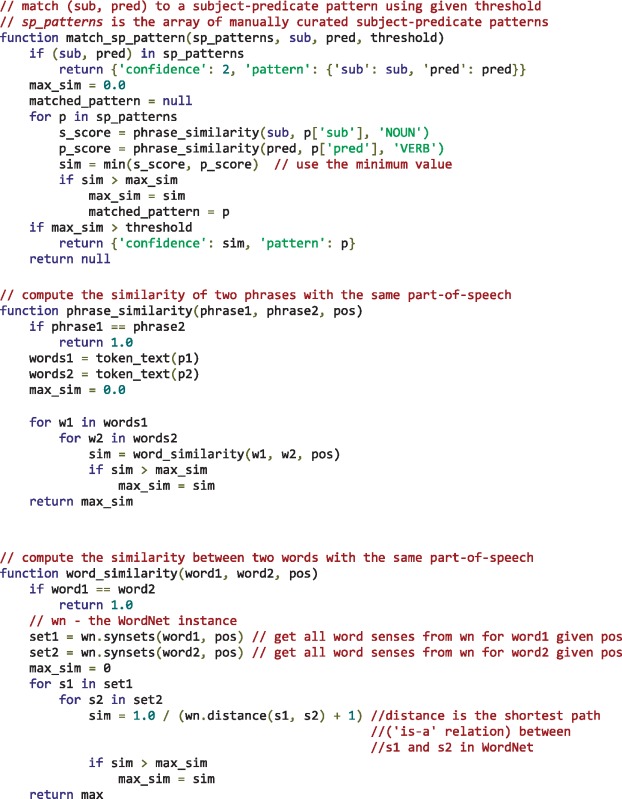

To consider such factors, we included two different spatial features in our algorithm: (i) sequential regions and (ii) the structure of a paper. Sequential regions were obtained by proportionally splitting the sentences of a paper into five ordered regions. Each sentence was assigned to one of these five regions only, based on its position in the paper. For example, all sentences into the first 20% of the paper was assigned the label ‘r0’, those falling into the consecutive 20% was labelled as ‘r1’, and so on.

The structure of article was incorporated into the algorithm by utilising the section title of the section a sentence falls into. While these section titles could be extracted from PDF files directly using the Poppler Qt4 library, we opted for those section types that were assigned by Partridge when converting the PDF file to XML. This decision was taken for two reasons. Firstly, this information is available without any further processing as a result from the PDF conversion. Secondly, Partridge section names are based a consistent terminology (e.g. introduction, methods, discussion, etc.), which makes this feature comparable across all papers.

Figure 2 illustrates the spatial distribution of highlighted goal sentences from the 183 papers contained in the development collection. This figure shows that goal sentences tend to appear in the top 30% of all sentences, while most of them reside within sections of type Introduction, Methods, or Other. These observations confirm the correlation between spatial features and highlighted sentences. We note here that the section type Other is predominantly assigned to sentences from the introduction of the paper (manually investigated, results not shown), but the tool used for PDF conversion failed to recognise the section in its entirety. The spatial distribution of all types of highlighted sentences is included in supplementary documents 2 and 3, which is also available online (https://plot.ly/∼honghan.wu/24/goal-method-findings-general/).

### Deriving a sentence-based score to determine relevant sentences

#### Weighing language patterns

Given a language pattern p (named entity, cardinal number, and subject-predicate pair), we calculated its importance in highlight prediction by using Equation 1, where $R_{HT}\left(p\right)=\frac{\#Highlighted\;sentences\;with\;p}{\#All\;highlighted\;sentences}$ is the percentage of highlighted sentences where p appears. Similarly, $R_{NH}\left(p\right)$ is the percentage of sentences that are not highlighted but contain the language pattern p. A threshold ε (in our Case 0.015) is defined to regulate the weight function to prevent unwanted high values of rare patterns. Our threshold of 0.015 was chosen from the setting that led to the best macro-averaged F_1_-measure on the development data. The value of ε is likely to be corpus dependent and should be interpolated from a reasonably sized corpus.

(1) $$w\left(p\right)={log}_{2}\frac{R_{HT}\left(p\right)+\varepsilon }{R_{NH}\left(p\right)+\varepsilon }$$

#### Using spatial information in weights

Considering the different spatial regions of a paper, a language pattern p could have multiple weights, i.e. one weight value per region. A spatial region is an area in a two-dimensional space identified by the two features introduced in Section 2.3: sequential regions and structure dimension. Intuitively, each cell in the matrix in Figure 2 could represent one region. Given a region r, the weight of p is then defined as $w\left(p,r\right)={log}_{2}\frac{R_{HT}\left(p,r\right)+\varepsilon }{R_{NH}\left(p,r\right)+\varepsilon }$ where $R_{HT}\left(p,r\right)=\frac{\#Highlighted\;sentences\;with\;p\;in\;region\;r}{\#Highlighted\;sentences\;in\;region\;r}$ and $R_{NH}\left(p,r\right)$ is the fraction of sentences with p that are not highlighted.

#### Overall scoring with spatial boosting

For a sentence $\hat{s}$ in region r, the overall score of $\hat{s}$ is calculated using equation 2, where CDs is the set of nouns with cardinal numbers; NEs is the set of named entity patterns; sp is the subject-predicate pattern; α, β, and γ are weights assigned to components of the three language patterns and α + β + γ = 1. We conducted a parameter scan and chose the values that led to the best macro-averaged F_1_-measure on the development data. b(sp, r) is a boosting function that further weighs a sentence based on its spatial location and type (subject-predicate pattern) as defined in Equation 3. In this function, R is the set of all regions defined in the corpus and k is a constant value to regulate how quick the overall score decreases when the (region- and type-based) frequency decreases.

(2) $$S(\hat{s},r)=\;(\alpha \;\times \sum_{C_{i}\in CDs}w(c_{i},r)+\beta \times \sum_{n_{i}\in NEs}w\left(n_{i},r\right)+\gamma \times w(sp,r))\times b(sp,r)$$

(3) $$b\left(sp,r\right)={\left(\frac{\#Highlighted\;sentences\;with\;pattern\;sp\;in\;region\;r}{\underset{\forall r_{i}\in R}{\max}\#Highlighted\;sentences\;with\;sp\;in\;r_{i}}\right)}^{k}$$

#### An example of language pattern scoring for a sentence

**Figure 3.**
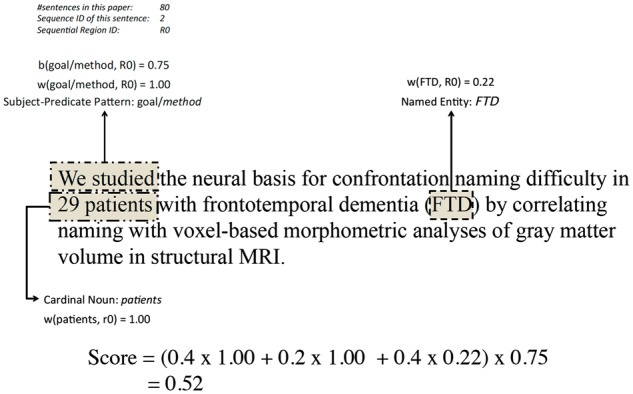
Sentence illustration of scoring and final result.

Figure 3 illustrates an example of how a sentence is scored based on the aforementioned features and weighing/scoring functions. The top-left corner shows the length of the paper from which the sentence was extracted (here 80 sentences), the sequential ID of the sentence (2) and the region ID that the sentence belongs to (r0). The subject-predicate pattern is ‘we studied’, which was categorised as a goal/method pattern by the curator. The weight of this pattern is 1.00 when it appears in the first sequential region (r0). The cardinal noun pattern ‘29 patients’ is identified and also weighed as 1.00 when it appears in r0. The named entity ‘FTD’ is identified in the sentence with a weight of 0.22 in this region. Finally, with subject-predicate pattern being a goal/method pattern in r0, the boosting function returns 0.75 (based on the statistics of the development data set). Using the settings of α = 0.4, β = 0.2, γ = 0.4, the final score is calculated to be 0.52.

### Evaluation methods of the results

#### Performance metrics for automatic highlights

We assessed our method to automatically determine PDF highlights for curation purposes with an automated and a manual evaluation. For the automated assessment, we calculated micro- and macro-averages for both the development and test data set (see 2.1 for more details on data sets) for precision, recall and F_1_-measure. The equations for each of the measures are as follows.

$$precision=\frac{TP}{TP+FP}$$

$$recall=\frac{TP}{TP+FN}$$

$$F_{1}-measure=\frac{2*precision*recall}{precision+recall}$$

In the manually highlighted papers, there are sentences that were only partially highlighted, e.g. one or two words. In such cases, we considered the entire sentence as highlighted because sentences are the basic units in our model. We note here that if a sentence contains more than one curated highlight, this sentence is counted only once for the performance assessment. Sentences that have been identified as relevant by the algorithm but contained no highlight from the curator were classified as false positives (FP) and sentences that contained a highlight by the curator but have not been automatically identified as relevant have been classified as false negatives (FN). The macro-average is determined by calculating precision, recall and F_1_-measure for each individual paper in the collection first and then averaging across all the papers in the data set. The micro-average is calculated by taking the same measures and determining TP, FP and FN across the entire collection of papers instead of on a per paper basis first.

In addition to the automated performance assessment, we conducted a manual analysis (author AO) of 22 papers (3404 sentences, 448 extracted as highlighted by curator, 421 determined as relevant by method) of the evaluation data set to identify causes for highlights that have been missed or added in addition, when automatically marking relevant sentences of a PDF. We grouped these errors according to the features involved in determining the relevance of a sentence.

Furthermore, the manual evaluation also aimed at assessing the performance of the automated extraction of sentence highlights using the Poppler Qt4 Python library. For this purpose, the sentences extracted by the library were verified manually by comparison to the PDF highlights and corrected where necessary. In addition, sentences that have been automatically predicted were marked as to whether they refine content that has been highlighted earlier, e.g. if a highlighted sentence describes the cohort of the study and the disease condition, and a predicted sentence would define how the disease condition was assessed, then this sentence was marked as refining an earlier highlight. We used these corrected sentence highlights and refinement markers to calculate an updated performance measure on the twenty-two papers in the reduced data set.

#### Comparison to bag-of-words baseline models

To evaluate the performance of the proposed methodology, we implemented various binary classifiers based on a bag-of-words model as baselines. Specifically, the problem was viewed as a binary classification problem where sentences were the data items to be labelled either as ‘highlight’ or as ‘normal’. Each sentence was characterised as a bag-of-words. This means, technically (after removing stop words) a sentence was abstracted as a vector, where each component represented a word and its value was a tf-idf (term frequency–inverse document frequency) score. Using such vectors as inputs, four well-known binary classification algorithms were adopted to classify sentences: Perceptron (36), Passive Aggressive Classifier (37), kNN (38) and Random Forest (39). Their performances were assessed using the same metrics on the test data set, which were compared and reported in the result section.

#### User based evaluation in a knowledge curation task

In addition to the automated evaluation experiments, we designed and conducted a user-based evaluation. The curator assigning the highlights to the PDFs was tasked to assess the usefulness of the automatically generated highlights in facilitating the knowledge curation tasks of the ApiNATOMY project (as described in Introduction). Forty unseen publications were selected and automatically highlighted. A web-based user interface (http://napeasy.org/exp/napeasy_eval.html) was developed to collect the human curator’s assessment on these highlights. Before starting the evaluation, the human curator was informed about the curation task and evaluation scenario. During the experiment, the curator worked on one publication at a time. The system presented the highlighted sentences and their categories. It also allowed the curator to reveal the full text of the publication, which was hidden by default. After assessing the highlighted sentences, the curator was asked to answer the following five questions before moving to the next publication (if there is still any). For Q1 and Q5, the answer was based on a scale of 1–5, which ranged from strongly disagree(1) to strongly agree(5).

- Q1: The highlights form a good representation of the study. (a scale of 1–5)
- Q2: The highlights are sufficient for you to extract correlation(s) between neuropsychometric tests and brain regions. (yes/no)
- Q3: The highlights contain irrelevant or not very helpful sentences (e.g., background information, two fine-grained details). (yes/no)
- Q4: There are too many highlighted sentences. (yes/no)
- Q5: The highlights contain sufficient provenance information for the abstracted correlation(s). (a scale of 1–5)

The questionnaire results of all forty papers were aggregated together to form the result of this extrinsic evaluation.

## Results

### Subject–predicate pairs used in automated highlighting procedure

In order to automatically determine relevant sentences in papers, we used a variety of linguistic and spatial features to determine whether a sentence needs to be highlighted or not. One of these features was the subject-predicate pairs that had been extracted from curator highlighted sentences and further classified into three categories (see Section 2.2 for more details). From the 1931 highlighted sentences in the development data set, we extracted 1427 subject-predicate pairs with 133 indicating a goal, 804 characterising a method and 579 suggesting a finding in the corresponding sentence. 257 subject–predicate pairs could not be assigned to any of the three categories as either the subject/predicate were missing or incorrectly extracted due to issues with the POS tagging output.

Subject–predicate pairs falling into the category ‘goal’ were typically expressed using subjects like ‘aims’, ‘goal’ or ‘we’; or predicates such as ‘intended’, ‘studied’, ‘aimed’ or ‘sought’. In most cases, subject–predicate pairs expressing a goal were also classified as expressing a method. For example, a sentence similar to ‘We assessed the brain volume in order to identify diseased individuals.’, the subject (We) and the predicate (assessed) could indicate a goal as well as method. Subject-predicate pairs alluding to ‘methods’ contained (among others) descriptions about study objects (subjects: ‘patients’, ‘participant’ or ‘subjects’; predicates: ‘required’, ‘asked’ or ‘stimulated’) or data collection (subjects: ‘time’, ‘MR images’, or ‘examinations’; predicate: ‘acquired’, ‘measured’ or ‘registered’). Pairs likely to suggest ‘findings’ were for example ‘our results show’ or ‘surface maps revealed’. A complete list of all subject–predicate pairs together with their categorisation can be accessed online (https://github.com/KHP-Informatics/napeasy/blob/master/resources/sub_pred_categories.json).

To assess how useful the correctly identified subject–predicate language patterns are in helping automated highlighting, we conducted an A/B testing on the subject–predicate feature—an experiment on two settings: one with and the other without the feature. On the development data set, the testing revealed that removing subject-predicate patterns led to a drop of the F1-measure from 0.52 to 0.30. This evaluation only assessed the contribution of correctly identified subject–predicate patterns. When the dataset changes to test data, the contribution might decrease due to the missing and/or incorrectly identified patterns.

In addition to the performance boosting, the subject-predicate pattern also enabled the sentence categorisation—assigning sentences to one or more categories of *goal*, *method* and *finding*, which is useful to speed up the curation tasks.

### Performance on automatically extracted highlights

Using linguistic and spatial features for the recognition of sentences relevant to describing relationships between neuropsychometric test and anatomy, led to the highlight of 1774, 1262 and 421 sentences in the development test and corrected test data sets respectively. Table 1 shows the obtained performance measures for the automated highlighting algorithm calculated as macro- and micro-measures. Performance measures for individual papers as required for the macro-average are available in the results section (https://github.com/KHP-Informatics/NapEasy/tree/master/results) of our online repository.

**Table 1.** Performance results of automated PDF highlights as obtained by described methodology

		Micro-average			Macro-average		
Dataset	Corr^a^	Precision	Recall	F_1_-measure	Precision	Recall	F_1_-measure
Development	no	0.50	0.53	0.52	0.51	0.54	0.53
Test	no	0.25	0.38	0.30	0.28	0.39	0.32
test_corrected	yes	0.50	0.47	0.49	0.53	0.49	0.51

As articles originate from different journals and differ in length and highlights, we report both micro- and macro-averaged performance measures for all the assessed data sets (*development*, *test* and *test_corrected*).

a Signifies whether data set has been manually corrected before calculating the measures.

Using the development data set for evaluation, we achieved a macro F_1_-measure of 0.53 and a micro F_1_-measure of 0.52. Moving to the test data for which the subject–predicate patterns are likely to be only partially known, the performance of our method dropped to a macro F_1_-measure of 0.32 (micro F_1_-measure of 0.30). However, as this evaluation technique relies on the automated recognition of PDF highlights made by the curator, we manually verified highlights made to the PDFs as extracted by the Poppler Qt4 library (see Section 2.5). Using the corrected highlights and refinement, the performance increased to a respectable macro-averaged F_1_-measure of 0.51. We note here, that F_1_-measures vary greatly [see detailed results (https://github.com/KHP-Informatics/NapEasy/blob/master/results/macro_results.tsv)] between different papers and further assessment is required as to what causes these differences other than those identified through manual evaluation.

As for the baselines, the four bag-of-words classifiers were trained on development data and evaluated on the test data. Overall, the best F_1_-measure of 0.22 was achieved by the Perceptron and Passive Aggressive Classifier, while the other two classifiers achieved lower F_1_-measures due to poor recall performances. Table 2 reports the performance details of all baseline approaches.

**Table 2.** Performance results of bag-of-words baseline approaches: perceptron, passive aggressive classifier, kNN and random forest are four well-known binary classification algorithms; ‘test’ is the test data set containing 58 papers; ‘test_corrected’ is a set of 22 papers, of which PDF processing errors were corrected; best performances are highlighted in the table as bold values

Algorithm	Test			test_corrected		
	Precision	Recall	F_1_-measure	Precision	Recall	F_1_-measure
Perceptron	0.23	0.20	**0.22**	0.26	0.19	**0.22**
Passive aggressive classifier	0.27	0.18	**0.22**	0.28	0.16	0.21
kNN	0.80	0.01	0.02	0.80	0.01	0.02
Random forest	0.63	0.04	0.08	0.69	0.04	0.08

### Result of user based evaluation

**Figure 4.**
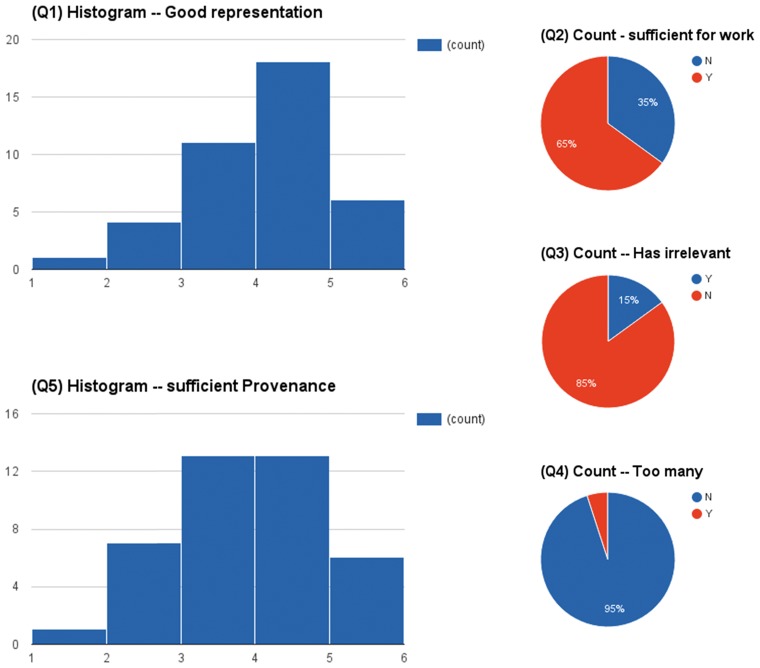
The assessment results of a user based evaluation in a scenario of supporting knowledge curation.

Figure 4 shows the aggregated results of the questionnaires on 40 papers. In summary, the highlights were concise in most cases (95% No to Q4) and only 15% cases contained some irrelevant information (Q3). In 65% cases (Q2), the highlights were sufficient enough to derive the sought-after relationships between brain structures and their functions in neurodegeneration. Almost half of the highlights (Q5) even provided sufficient provenance of conclusions of described studies.

### Evaluation identifies several limitations of generalised tools

To further assess the performance of our predictions and identify areas for future improvements, we conducted a manual assessment of 22 papers from the test data set. The manual assessment elucidated a variety of limitations impacting the performance of the algorithm suggested here.

*Missed semantic information*. Despite the broad range of annotations covered by the NCBO annotator and the NLTK named entity recogniser, semantic information is missed that could potentially improve the recognition of those sentences that are missed at the moment. In particular, semantic information on specific concepts used to refer to neuroanatomy and related tests are not covered in either tool. Another reason for missing semantic information is the extensive use of abbreviations in the full text of a paper, which are also not reliably recognised by the tools employed here.

*Cardinal numbers*. Furthermore, the approach chosen to determine cardinal numbers in conjunction with nouns has its limitations in that the Stanford parser does not label numbers given as words (e.g. ‘forty-five’ instead of ‘45’) with CD and uses JJ instead. Another issue with cardinal numbers is that they are used in different contexts (e.g. age ranges or cohort sizes), which if recognised could lead to an increased number of incorrectly predicted sentence highlights. This clearly shows that the simple approach we chose as a starting point needs replacing in subsequent iterations of the tool. We have not considered any alternative approaches to date.

*Recognition and use of subject–predicate pairs.* Unsurprisingly, the subject-predicate pair list automatically gathered from the development data set does not cover all the subject-predicate pairs used in the test data set. Furthermore, other subject–predicate pairs are too general, which can lead to false positives. For example, the phrase ‘the data revealed’ can be used in both circumstances when referring to one’s own work or when referring to work conducted by others, but highlighted sentences typically only cover those sentences that report about work conducted by the author(s) of the paper.

Sentence boundaries and ordering. In order for the spatial features to work properly, an exact recognition of sentence boundaries and their ordering is required. However, the manual analysis identified issues not only with the sentence boundary detection (e.g. sentences are merged together or mixed across columns), but also with the ordering of the sentences as assigned during the process of converting the PDF to an XML file.

## Discussion

In our study, we developed an algorithm to identify sentences that are relevant for a curator to be able to infer research goals, patient sample, neuroimaging techniques, neuropsychometric test and significant results correlating neuroanatomy and behavioural, cognitive or motor deficits. Using semantic, linguistic and spatial features, we were able to achieve a macro F_1_-measure of 0.51 when compared to manually curated highlights. Especially, the subject–predicate patterns and their categorisation enable customisable highlights in terms of skewing highlights towards certain types of sentences. Such a feature has been found useful in helping the curation tasks, e.g. by prioritising sentences pertaining to the main findings of the study. The highlights would give the human curator a quick understanding about whether a paper is worth further investigation or not. While these automated highlights could already be used for the purpose of providing sentences to curator, there are areas of improvement in future work.

Using the approach described, we achieved a higher performance when using the data set from which the subject–predicate pairs had been collected. The drop in performance when moving to the test data set is likely to be influenced by the nature and content of the papers. One aspect is that the subject–predicate pairs gathered from one data set are not sufficiently represented in the other and the synonyms provided in WordNet are not sufficient for a mapping. Other aspects contributing to this could be a change in either the type of the papers highlighted (review, experimental research or case study), the content of the paper or the journal issuing the publication. One possibility for refinement in future work is the derivation of a small set of universal subject-predicate pairs and the use of these as seed patterns for the extraction task. The categorisation into goal, method and finding would then have to be applied to the universal subject–predicate pairs and propagated to those patterns identified automatically.

As identified by the manual assessment, the tools and configurations thereof are not ideal. For now, all the tools have been used ‘off the shelf’ and no comparison with other configurations or tools has been conducted. For example, while the NCBO annotator can provide ontology annotations for all ontologies contained in BioPortal, as long as the ontologies do not include the vocabulary needed for the task at hand, performance will stay low. Furthermore, the selection of tools will need to be extended so that abbreviations (e.g. with (40) or (41)) can be extended to their full spelling before a semantic assessment happens which is likely to increase the overall performance due to higher rate of semantic features being available.

In addition to the aforementioned restrictions on performance, the current spatial feature implementation potentially limits the correct identification of highlights needed in a PDF for curation purposes. The way this feature was implemented is that it uses a sequential separation of the paper into regions. However, as we are covering three different types of publications (reviews, case studies and experimental research papers), the discourse of the article is likely to differ (19). Another factor likely to influence the performance of our spatial feature implementation is the coverage of a large number of journals. As journals impose their own formatting guidelines, the ordering may not be the same across all the papers investigated here. For example, one journal may require the ordering according to Introduction, Method, Result and Discussion, while another requests an ordering into Introduction, Results, Discussion and Method. As our manually curated data set is small in size, we saw no possibility to separate according to journal and still gather meaningful results that would allow us to draw conclusions. However, in future work we aim to explore this possibility to improve our highlighting algorithm by developing models for groups of journals instead of one universal one.

Finally, we acknowledge the limitation that the size of datasets used in this study was relatively small. But the primary value of this work comes from the unique approach to assisting curation, which might not necessarily be devalued by this limitation substantially.

## Conclusion

In our study, we aimed to extract sentences that could ease the curation of data relevant to neurodegenerative diseases such as Parkinson’s and Alzheimer’s disease. We employed semantics, subject–predicate pairs and spatial features to determine the relevance of a sentence, without setting any fixed cut-off values for the number of sentences highlighted in a paper. Using our approach, we achieved a macro F_1_-measure of 0.51 on an ‘unseen’ data set, after correcting for imprecision of the automated detection of curator highlights in PDFs. To evaluate the usefulness of the automatic highlights, we conducted an extrinsic evaluation on 40 new publications. The results showed that (a) the highlights were concise in most case (there was no irrelevant information in 85% of the cases); (b) in 65% of cases, the highlights were sufficient for the curator to conduct the curation task in a way that there was no need to consult the full text of publications. The result indicates that the proposed model can be trained on a curator’s own data and then be used to help speed up the curation work in most cases. While we could identify areas for further improvement by conducting a manual evaluation, we believe that our results are a first step in the direction of reducing curation costs by providing automated highlights for a specific curation task.

## Supplementary data

Supplementary data are available at *Database* Online.

## Supplementary Material

Supplementary Data
