# Supplementary material for: Automated PDF highlighting to support faster curation of literature for Parkinson’s and Alzheimer’s disease
Source: Database (Oxford). 2017 Mar 27;2017:bax027. doi: 10.1093/database/bax027 (PMC5467557; doi:10.1093/database/bax027)

**Supplementary Document 3**

The spatial distribution of ‘*methods*’ sentences extracted from the papers in the development data set


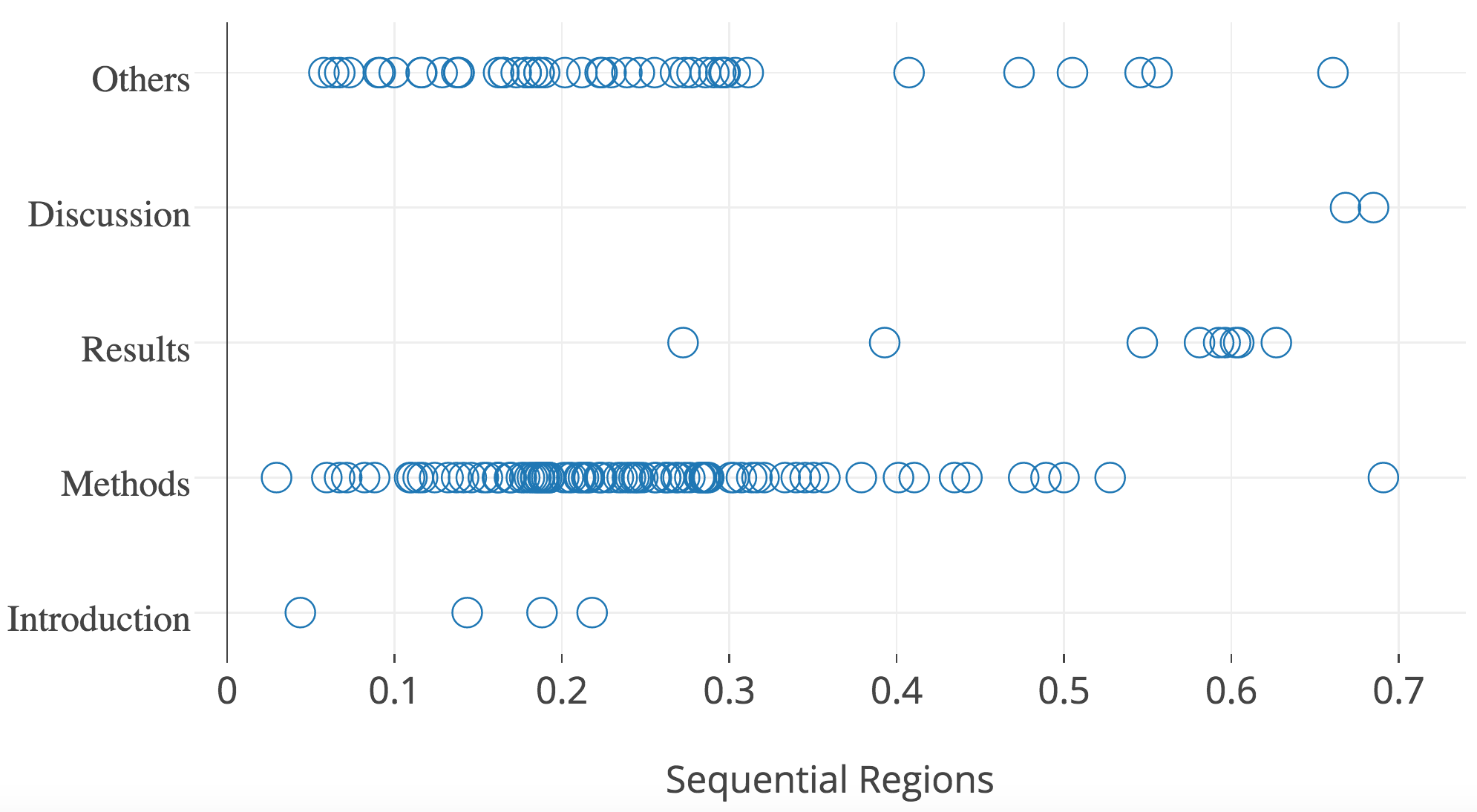

Supplement: Supplementary Data [file bax027_Supp.zip › sup3_methods_spatial.docx]
