# Supplementary material for: Automated PDF highlighting to support faster curation of literature for Parkinson’s and Alzheimer’s disease
Source: Database (Oxford). 2017 Mar 27;2017:bax027. doi: 10.1093/database/bax027 (PMC5467557; doi:10.1093/database/bax027)
Supplement: Supplementary Data [file bax027_Supp.zip › Description of Manual Curation Process from Christine.docx]

**Supplementary Document 1**

Description of Manual Curation Process

Christine Girges

When reviewing papers, curators are asked to extract the following information to create a correlation. These items are usually combined to form a description of the study too.

1. Detailed aim or goal of the study (e.g., the present study aimed to investigate the neural basis of tremors in Parkinson’s disease [PD]).
2. Type of located measurement used including, but not limited to: MRI; voxel-based morphometry (VBM); functional MRI; resting-state functional MRI; positron emission tomography (PET); single photon emission computed tomography (SPECT); diffusion tensor imaging (DTI); repetitive transcranial magnetic stimulation (rTMS); deep brain stimulation (DBS); post mortem imaging; immunohistochemistry.
3. Regarding No.2, it is also helpful to know whether the paper was looking at the structure (volume) or function of brain areas – this information is usually provided in context regarding the used imaging technique (e.g., voxel-based morphometry was implemented to observe grey matter atrophy in PD patients with tremors)
4. Participant characteristics:
   - Which clinical populations were used? (e.g., patients with PD or Alzheimer’s disease). Healthy controls are also sometimes used to provide a control or comparative group).
   - Studies may also compare two groups of patients (e.g., PD patients with and without tremors).
   - Details on diagnosis (if available)
   - How many participants took part?
5. Were neuropsychometric tests used? E.g., MDS-UPDRS, Tremor Rating Scale, etc. A full list of neuropsychometric tests can be found in taxonomy document already provided.
6. Some studies include a task or experimental paradigm that is not standardized. These should also be noted.
7. Significant findings. For example;
   - Compared to the control group, PD patients with tremors showed atrophy in the supplementary motor area and middle frontal gyrus.
   - Compared to PD patients without tremor, those with tremors showed atrophy in the amygdala.

Each result should be documented as a new correlation. So, for this example; Aim: Tremors in PD (symptom) were investigated using VBM (measurement). The symptoms were evaluated using the MDS-UPDRS and a Tremor Rating Scale (neuropsychometric test). Results showed grey matter changes in supplementary motor area, middle frontal gyrus and amygdala (brain regions).
